# Supplementary material for: Effects of the Phosphodiesterase 10A Inhibitor MR1916 on Alcohol Self-Administration and Striatal Gene Expression in Post-Chronic Intermittent Ethanol-Exposed Rats
Source: Cells. 2024 Feb 9;13(4):321. doi: 10.3390/cells13040321 (PMC10886814; doi:10.3390/cells13040321)
Supplement: Supplementary file 1 [file cells-13-00321-s001.zip › cells-2740536-supplementary.pdf]

Supplementary Material

1 Supplementary Figures

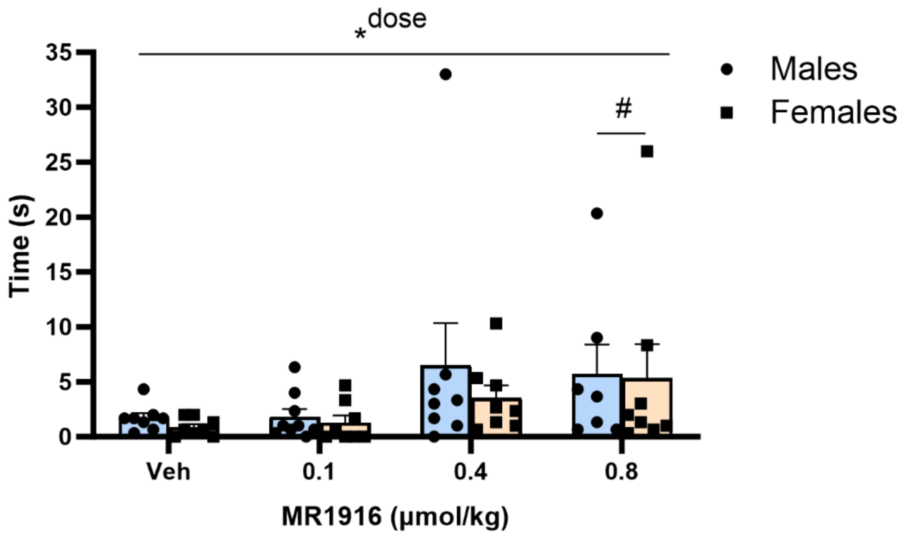

Figure S1. Effects of MR1916 pretreatment (-60 min) on the duration of cataleptic responses in male and female rats (n = 7-9/sex/dose) in the bar test. Histograms show group M+SEM, and scatter shows the scores of individual subjects averaged across 3 trials. *ANOVA Dose main effect. # and line differs significantly from vehicle (LSD test) (all $p < 0.05$).

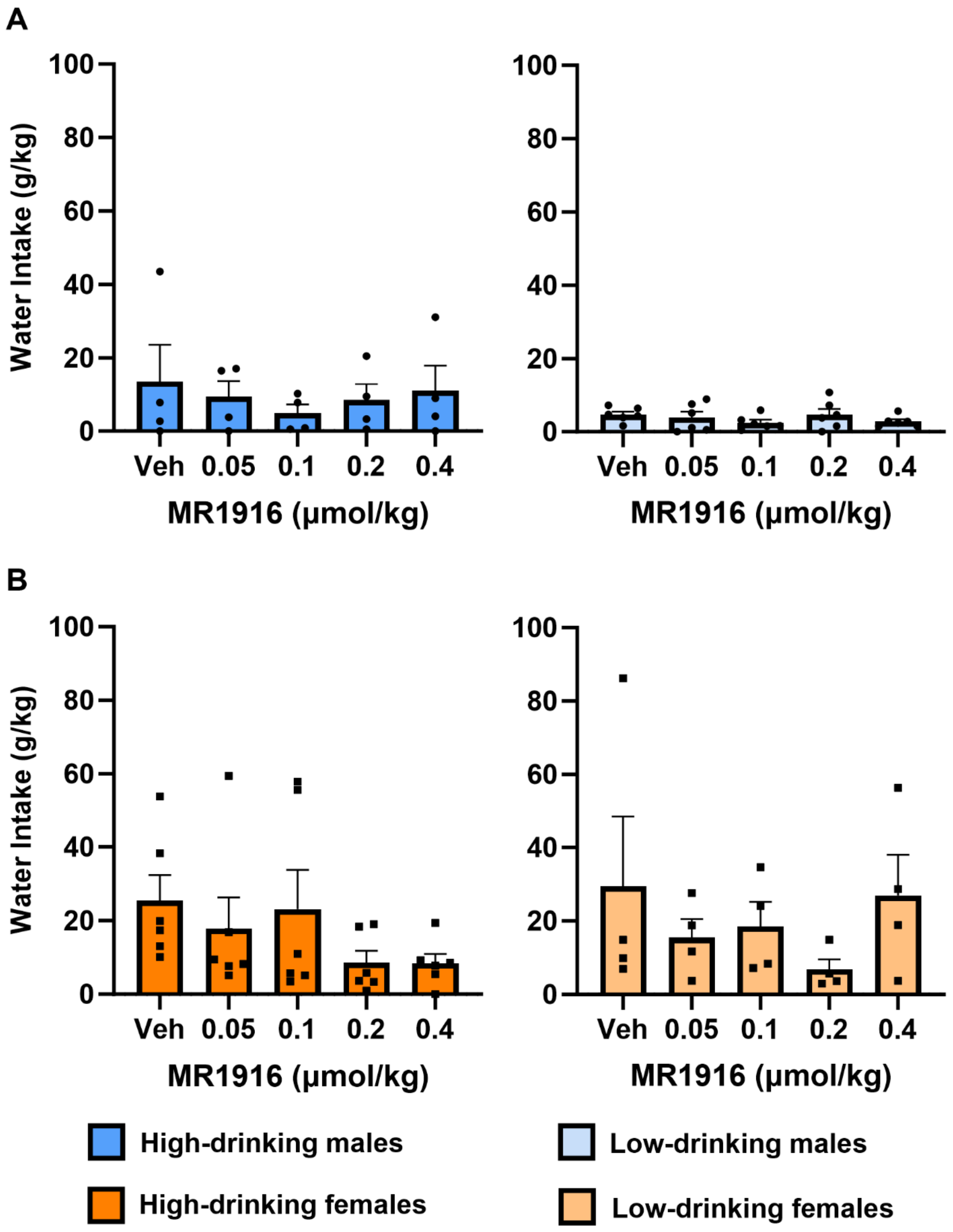

Figure S2. Effects of MR1916 pretreatment (-60 min) on water intake in post-CIE (A) male and (B) female rats on ethanol vs. water self-administration. Left panels show high-drinking rats, right panels show low-drinking rats. Histograms show group *M*+SEM, and scatter shows the scores of individual subjects. N = 10/sex.

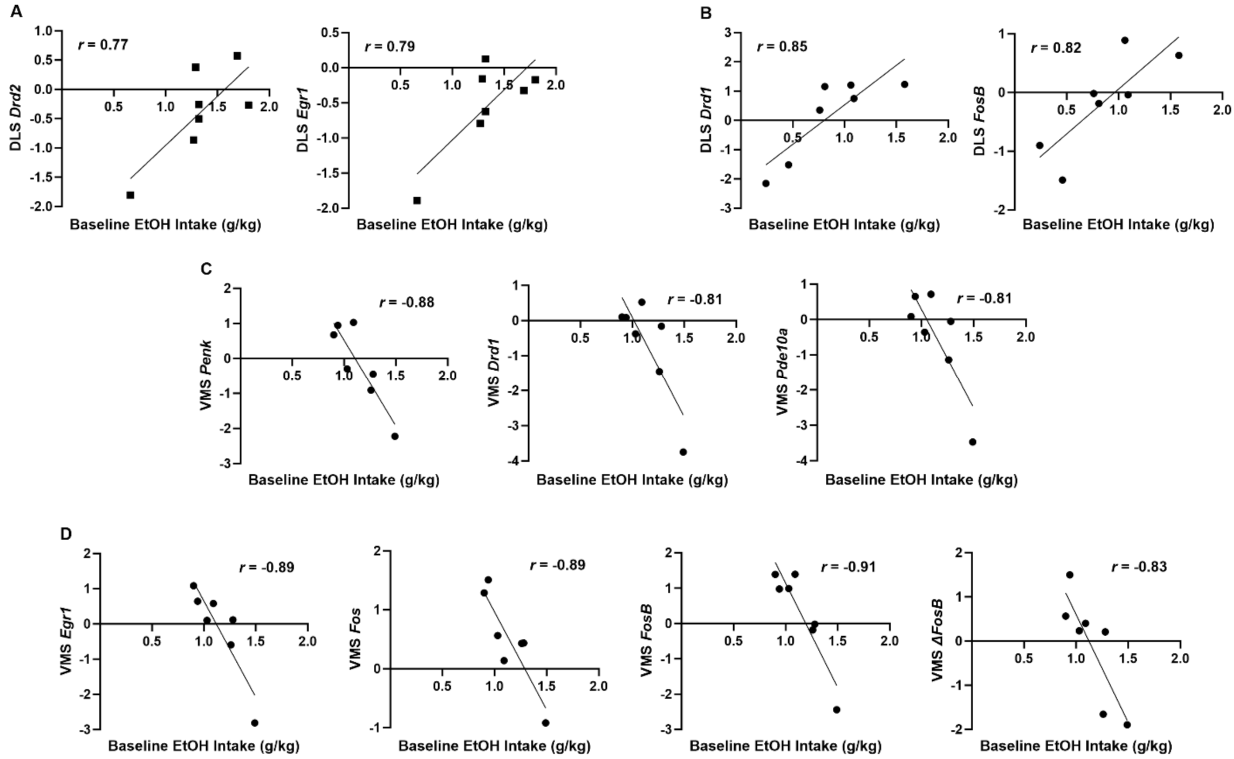

Figure S3. Pearson correlations between average baseline drinking and (A) dopamine receptor 2 (*Drd2* - left panel), and early growth response protein 1 (*Egr1* - right panel) expression in the DLS of post-CIE female rats pretreated with the vehicle control; (B) dopamine receptor 1 (*Drd1* - left panel), and FBJ murine osteosarcoma viral oncogene homolog B (*FosB* - right panel) expression in the DLS of male rats pretreated with 0.4 umol/kg MR1916; (C) enkephalin (*Penk* - left panel), dopamine receptor 1 (*Drd1* - middle panel), and phosphodiesterase 10a (*Pde10a* - right panel) expression in the VMS of female rats pretreated with 0.4 umol/kg MR1916; (D) early growth response protein 1 (*Egr1* – far left panel), FBJ murine osteosarcoma viral oncogene homolog (*Fos* – middle-left panel), FBJ murine osteosarcoma viral oncogene homolog B (*FosB* – middle-right panel), delta FBJ murine osteosarcoma viral oncogene homolog B (*ΔFosB* – far right panel) expression in the VMS of female rats pretreated with 0.4 umol/kg MR1916. N = 7.

2 Supplementary Tables

Table S1. Forward and reverse primer sequences used for qPCR.

Primer	Primer Sequence	Accession number
Ywhaz [74]	FP – 5′ – GAA AAT GAA GGG TGA CTA CTA C – 3′<br>RP – 5′ – CTG ATT TCA AAT GCT TCT TGG – 3′	NM_013011.4
Ppia [75]	FP – 5′ – TTT GGG AAG GTG AAA GAA GGC – 3′<br>RP – 5′ – ACA GAA GGA ATG GTT TGA TGG G – 3′	NM_017101.1
Tac1 [76]	FP - 5' – ACG CAC TAT CTA TTC ATC TTC ATC - 3'<br>RP - 5' – AGA ATT ACA AGG CTT ATT GGC A – 3′	NM_012666.2
Drd1 [77]	FP - 5' - GAA GCA AAT CCG GCG CAT CTC - 3'<br>RP - 5' - TTC AGA CTG GGC GCA TTC GAC - 3'	NM_012546.3
Drd2 [77]	FP - 5' - CCT TAA GAC GAT GAG CCG CAG AA - 3'<br>RP - 5' - GGT TGA CGG CAC TGT TGA CAT AGC - 3'	NM_012547.3
*Penk**	FP – 5′ – ACT TCC TGG CAT GCA CAC TCG AA– 3′<br>RP - 5' – CGA TGT TAT CCC AAG GGA ACT CGG– 3′	NM_017139.2
*Pde10a**	FP – 5′ – CGC CAC TGA CCT CGC ACT GT – 3′<br>RP – 5′ – CGC AGG CAG TCA TCA TCA AGC – 3′	NM_022236.2
Egr1 [78]	FP - 5' - TGC ACC CAC CTT TCC TAC TC - 3'<br>RP - 5' - AGG TCT CCC TGT TGT TGT GG - 3'	NM_012551.3
Fos [79]	FP - 5' - CAG CCT TTC CTA CTA CCA TTC C - 3'<br>RP - 5' - ACA GAT CTG CGC AAA AGT CC - 3'	NM_022197.2
FosB [47]	FP - 5' - GTG AGA GAT TTG CCA GGG TC - 3'<br>RP - 5' - AGA GAG AAG CCG TCA GGT TG - 3'	NM_001256509.1
ΔFosB [47]	FP - 5' - AGG CAG AGC TGG AGT CGG AGA T - 3'<br>RP - 5' - GCC GAG GAC TTG AAC TTC ACT CG - 3'	XM_039101873.1

* Primers designed *in house.*

Table S2. *F*-statistic and *Dfs* for Anova analysis

Variable	Grouping	Type of effect	Effect	df1	df2	F-ratio	p-value
Ethanol intake	Overall	Between	drinking level	1	16	6.655	0.02
		Within	dose*drinking level	4	64	2.746	0.036
		Quadratic contrast[1]	dose	1	16	10.96	0.004
	Males	Linear contrast[1]	dose	1	8	6.93	0.03
		Linear contrast[1]	dose*drinking level	1	8	10.367	0.012
	Females	Quadratic contrast[1]	dose	1	8	7.873	0.023
		Between	drinking level	1	16	6.655	0.02
	High drinking females	Quadratic contrast[1]	dose	1	5	21.139	0.006
	Low drinking males	Linear contrast[1]	dose	1	5	9.599	0.027
Ethanol preference	Overall	Between	sex	1	16	5.073	0.039
		Quadratic contrast[1]	dose	1	16	19.695	<0.001
	Females	Linear contrast[1]	dose*drinking level	1	8	10.367	0.012
	High drinking females	Quadratic contrast[1]	dose	1	5	8.402	0.034
	Low drinking females	Within	dose	4	12	3.343	0.047
		Quadratic contrast[1]	dose	1	3	14.342	0.031
Water intake	Overall	Between	sex	1	16	7.552	0.014
Drd1	Naïve – DLS	Between	treatment	1	19	4.955	0.038
Drd2			treatment	1	19	5.467	0.03
Pde10a			treatment	1	19	5.477	0.03
			sex	1	19	10.245	0.005
Egr1			treatment	1	19	27.001	<0.001
			sex	1	19	13.984	0.001
Fos (ln)			sex	1	19	5.351	0.032
FosB			treatment	1	19	13.425	0.002
			sex	1	19	51.639	<0.001
ΔFosB			treatment	1	19	7.587	0.013
			sex	1	19	25.007	<0.001
Tac1	Naïve - VMS	Between	sex	1	19	5.3	0.033
Drd1			sex	1	19	4.895	0.039
Drd2			sex	1	19	35.054	<0.001
Pde10a			sex	1	19	19.508	<0.001
Egr1			sex	1	19	15.588	0.001
FosB			treatment*sex	1	19	5.838	0.026
			treatment	1	19	4.736	0.042

			sex	1	19	16.205	0.001
ΔFosB			sex	1	19	8.505	0.009
Penk	Post-dependent - DLS	Between	treatment	2	35	3.477	0.042
			sex	1	35	5.702	0.008
Egr1			treatment	2	35	8.725	0.001
Fos			treatment*sex	2	35	7.817	0.002
			treatment	2	35	16.941	<0.001
			sex	1	35	5.186	0.029
FosB			treatment*sex	2	35	4.263	0.022
			treatment	2	35	8.625	0.001
			sex	1	35	11.82	0.002
ΔFosB			treatment	2	35	4.274	0.022
Fos	Post-CIE - VMS	Between	treatment	1	35	7.239	0.002
Tac1	High vs. Low drinking male rats - DLS	Between	treatment*drinking level	2	16	10.365	0.001
Drd1			treatment*drinking level	2	16	6.629	0.008
			drinking level	1	16	6.045	0.026
Pde10a			treatment*drinking level	2	16	3.914	0.041
FosB			drinking level	1	16	6.571	0.021
Egr1	High vs. Low drinking male rats - VMS	Between	treatment*drinking level	2	15	9.156	0.003
			treatment	2	15	14.8	<0.001
			drinking level	1	15	5.669	0.031
Fos			treatment*drinking level	2	15	5.552	0.016
			treatment	2	15	4.601	0.028
ΔFosB			drinking level	1	15	6.369	0.023

**Variable**	**Grouping**	**Type of effect**	**Effect**	**Contrast Estimate**	**SE**	**95% CI (Lower-Upper)**	**p-value**
Penk	Post-dependent - DLS	Linear contrast[2]	treatment	0.588	0.224	0.133 - 1.043	0.013
Egr1				0.808	0.209	0.838 - 1.233	<0.001
Fos				0.942	0.175	0.587 - 1.298	<0.001
FosB				0.755	0.198	0.352 - 1.157	0.001
ΔFosB				0.429	0.182	0.058 - 0.799	0.024

Fos	Post-dependent - VMS	Linear contrast[2]	treatment	0.792	0.221	0.343 - 1.240	0.001
Egr1	High vs. Low drinking male rats - VMS	Quadratic contrast[2]	treatment	0.684	0.167	0.328 - 1.039	0.001
Fos		Linear contrast[2]		0.773	0.3	0.133 - 1.414	0.021

[1]Within-subject contrast analysis. [2]Between-subject (K-matrix) contrast analysis.

3 References

47. Weiss, F., Lorang, M. T., Bloom, F. E., & Koob, G. F. (1993). Oral alcohol self-administration stimulates dopamine release in the rat nucleus accumbens: genetic and motivational determinants. Journal of Pharmacology and Experimental Therapeutics, 267(1), 250-258.
74. Oliveira, S. R., Vieira, H. L., & Duarte, C. B. (2015). Effect of carbon monoxide on gene expression in cerebrocortical astrocytes: Validation of reference genes for quantitative real-time PCR. Nitric Oxide, 49, 80-89.
75. Boué, J., Blanpied, C., Brousset, P., Vergnolle, N., & Dietrich, G. (2011). Endogenous opioid-mediated analgesia is dependent on adaptive T cell response in mice. The Journal of Immunology, 186(9), 5078-5084.
76. Valenza, M., Steardo, L., Cottone, P., & Sabino, V. (2015). Diet-induced obesity and diet-resistant rats: differences in the rewarding and anorectic effects of D-amphetamine. Psychopharmacology, 232(17), 3215-3226.
77. Burnett, L. A., Blais, E. M., Unadkat, J. D., Hille, B., Tilley, S. L., & Babcock, D. F. (2010). Testicular expression of Adora3i2 in Adora3 knockout mice reveals a role of mouse A3Ri2 and human A3Ri3 adenosine receptors in sperm. Journal of Biological Chemistry, 285(44), 33662-33670.
78. Kim, H. S., Suh, K. S., Sul, D., Kim, B. J., Lee, S. K., & Jung, W. W. (2012). The inhibitory effect and the molecular mechanism of glabridin on RANKL-induced osteoclastogenesis in RAW264. 7 cells. International journal of molecular medicine, 29(2), 169-177.
79. Alibhai, I. N. (2006). Regulation of FOSB MRNA Isoforms by Drugs of Abuse (Doctoral dissertation).
